# The Effect of Oral Semaglutide on Cardiovascular Risk Factors in Patients with Type 2 Diabetes: A Systematic Review

**DOI:** 10.3390/jcm14072239

**Published:** 2025-03-25

**Authors:** Stanislaus Ivanovich Krishnanda, Marie Christabelle, Oliver Emmanuel Yausep, Caroline Sugiharto, Leroy David Vincent, Raksheeth Agarwal, Ivan Damara, Dante Saksono Harbuwono

**Affiliations:** 1 Faculty of Medicine, Cipto Mangunkusumo National General Hospital, University of Indonesia, Jakarta 10430, Indonesia; marie_christ98@hotmail.com (M.C.); oliveremmanuel@hotmail.com (O.E.Y.); leroydavidvincent95@gmail.com (L.D.V.); 2 Faculty of Medicine, Universitas Pelita Harapan, Tangerang 15810, Indonesia; johannacarolines@gmail.com; 3 Internal Medicine at Jacobi Medical Center/Albert Einstein College of Medicine, Bronx, NY 10461, USA; raksheeth@hotmail.com; 4 Internal Medicine Department, Weiss Memorial Hospital, Chicago, IL 60640, USA; ivandamara27@gmail.com; 5 Division of Endocrinology, Metabolism, and Diabetes, Department of Internal Medicine, Faculty of Medicine, Cipto Mangunkusumo National General Hospital, Universitas Indonesia, Jakarta 10430, Indonesia

**Keywords:** GLP-1 receptor agonist, oral semaglutide, type 2 diabetes mellitus, cardiovascular, blood pressure, lipid, cholesterol

## Abstract

**Background/Objectives**: There has been a prominent rise in the use of GLP-1 RAs recently, particularly semaglutide, for the treatment of T2DM with or without obesity. Subcutaneous injections of semaglutide have demonstrated beneficial effects on cardiovascular risk factors. However, several factors hinder the use of subcutaneous administration. Therefore, the oral route is preferred; **yet**, it remains unclear whether oral semaglutide provides cardiovascular protection comparable to its subcutaneous counterpart. **Methods**: A systematic review in line with the PRISMA guidelines was performed based on eight databases (Scopus, Proquest, Science Direct, PubMed, Google Scholar, EBSCOHost, Clinical Key, and The Cochrane Library) to identify clinical studies that assessed the effects of oral semaglutide on cardiovascular risk factors, especially blood pressure and lipid or cholesterol profile in T2DM patients. Inclusion criteria included studies that used oral semaglutide on top of a mainstay treatment for T2DM compared to the placebo control group, assessed cardiovascular risk factors, and were conducted prospectively or in an RCT design. Case reports, ongoing studies with incomplete results, reviews, animal studies, and retrospective studies were excluded. The Newcastle-Ottawa scale and Jadad scale were used to assess the risk of bias in the included studies. Data extracted from the selected studies included patient characteristics, study design, research methodology, intervention regimen, and cardiovascular risk factors: SBP, DBP, TC, HDL, LDL, and TG. Data were presented in a table format to compare and synthesize the results of each study. **Results**: Five clinical studies were selected (two were randomized trials and three were observational, prospective studies). All five studies reported a consistent trend in the reduction in SBP (ranging from −2.60 to −12.74 mmHg) after oral semaglutide treatment. However, its effect on DBP was found to be less consistent. Lipid profile results show the most consistent trend in total cholesterol reduction (−8.80 to −22.19 mg/dL). Four studies reported a favorable reduction in LDL cholesterol (−7.6 to −18.0 mg/dL) and triglycerides (−11.00 to −40.13 mg/dL). HDL cholesterol shows the least consistent findings where three studies reported an increasing trend, yet this was not statistically significant; one study reported a mild increase in HDL (+0.90 ± 0.12; *p* < 0.0001); and one study reported a slight reduction in HDL (55.6 ± 2.5 to 51.6 ± 2.2; *p* < 0.05). **Conclusions**: Once-daily oral semaglutide is a promising add-on therapy for the treatment of T2DM with or without obesity in reducing cardiovascular risk factors, potentially lowering cardiovascular-related mortality. Thus, once-daily oral semaglutide may offer cardiovascular benefits comparable to the subcutaneous form, with the advantage of improved adherence.

## 1. Introduction

Patients with type 2 diabetes mellitus (T2DM) have a two- to six-fold increase in the risk of death from cardiovascular disease compared to patients without T2DM, wherein coronary artery disease accounts for more than 75% of all hospitalizations and 80% of all deaths in T2DM patients [1].

The treatment of T2DM includes various antihyperglycemic agents, cholesterol-lowering drugs, and insulin therapy. Of these, two drug classes have demonstrated the modification of cardiovascular risk, namely glucagon-like peptide-1 (GLP-1) receptor agonists and sodium-glucose cotransporter-2 (SGLT2) inhibitors [2].

GLP-1 receptor agonists (GLP-1RAs) lower HbA1c by enhancing glucose-dependent insulin secretion, while also reducing glucagon secretion, slowing gastric emptying, and decreasing appetite [3].

Treatment with GLP-1RAs has also demonstrated modest improvements in blood pressure as well as reductions in lipid levels and body weight, with a low risk of hypoglycemia. On the other hand, GLP-1RAs differ in structure and duration of action, leading to variability in their cardiovascular effects across studies [4,5,6,7,8].

Semaglutide is one such GLP-1RA that has been increasingly used in recent experimental treatment of T2DM with promising results on cardiovascular risk factors [4,9]. However, the traditional use of semaglutide via the subcutaneous route is not without challenges, particularly the issue of non-adherence [10,11]. Thus, an alternative way of administering the medication is important to improve adherence [12,13]. The oral route is demonstrated to have superior adherence to the traditional subcutaneous route [14]. Nevertheless, it is not yet clear whether oral administration of semaglutide displays similar cardiovascular benefits to that of its subcutaneous counterpart. A systematic review reported that oral semaglutide bioavailability was low, and its systemic exposure was relatively lower compared to the subcutaneous route [15]. This might potentially affect its efficacy, including its cardiovascular benefits. While data on glycemic improvement and weight loss effects were widely studied, oral semaglutide benefits on cardiovascular risk factors had just recently begun to be explored. PIONEER 6 [16] was one pivotal trial that explored the cardiovascular effect of oral semaglutide. The trial was mainly designed to explore the cardiovascular safety of oral semaglutide, while cardiovascular risk factors were not their focus. Thus, the question of whether oral formulation has benefits on cardiovascular risk factors is yet to be addressed. This systematic review aims to explore the potential benefits of oral semaglutide in addition to the mainstay treatment regimen on cardiovascular risk factors in patients with T2DM.

## 2. Materials and Methods

This systematic review was conducted following the guidelines set by the Preferred Reporting Items for Systematic Reviews and Meta-Analyses (PRISMA) 2020.

### 2.1. Search Strategy

A literature search was performed on 15 November 2024, across 8 databases (Scopus, Proquest, Science Direct, PubMed, Google Scholar, EBSCOHost, Clinical Key, and The Cochrane Library) to identify clinical studies that assessed the effects of oral semaglutide on cardiovascular risk factors in T2DM patients. The search terms used in each database were as follows: Oral semaglutide AND Diabetes AND Cardiovascular. Initially, the titles and abstracts of studies were screened. A full-text analysis of the selected articles was then conducted according to pre-established eligibility criteria. Additionally, the reference lists of included studies and relevant review articles were reviewed to identify additional potential studies. The screening and analysis were carried out independently by two researchers.

### 2.2. Eligibility Criteria

Our inclusion criteria included studies that used oral semaglutide on top of mainstay treatments for T2DM compared to the placebo control group, assessed cardiovascular risk factors, and were conducted prospectively or in a randomized controlled trial design, without time or language limitations. The following were excluded: case reports, ongoing studies with incomplete results, reviews, animal studies, and non-prospective or retrospective studies.

### 2.3. Data Extraction

The relevant data extracted from each study included patient characteristics, study design, research methodology, intervention regimen, and cardiovascular risk factors measures including systolic blood pressure (SBP), diastolic blood pressure (DBP), total cholesterol (TC), low-density lipoprotein (LDL), high-density lipoprotein (HDL) and triglycerides (TGs). Effect measures extracted include treatment difference, change from baseline, and ratio to baseline. All data were tabulated to compare and synthesize the results.

### 2.4. Quality Assessment

The quality of the included cohort studies was evaluated using the Newcastle-Ottawa Scale (NOS), which consists of 3 main components: selection of the study group, comparability, and outcomes. Studies rated as high quality received 3 or 4 stars in the selection component, 1 or 2 stars in the comparability component, and 3 stars in the outcome component. Studies of fair quality received 2 stars in the selection component, 1 or 2 stars in the comparability component, and 2 or 3 stars in the outcome component. Studies classified as poor quality received 0 or 1 star in the selection component, 0 stars in the comparability component, and 0 or 1 star in the outcome component. Meanwhile, the Jadad scale was used to evaluate the quality of randomized controlled trial studies. It evaluates whether the studies possess proper randomization (0–2 points given), blinding (0–2 points given), and information on patients withdrawn or dropped out from the study (0–1 point given).

## 3. Results

### 3.1. Search Results

**Figure 1:**
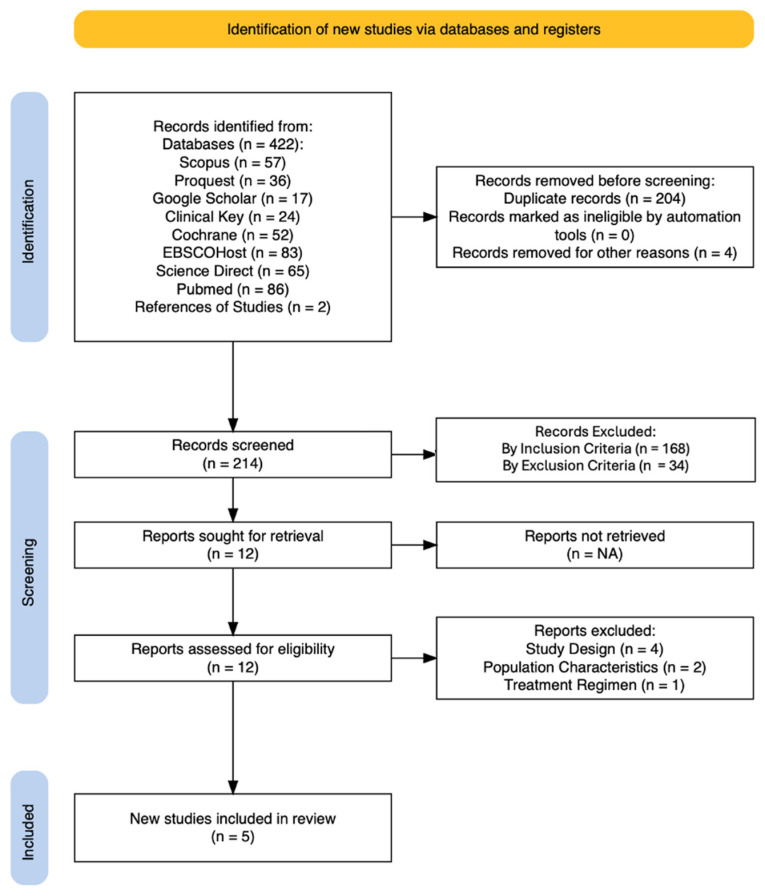
PRISMA flow diagram.

The literature search results are summarized in the form of a PRISMA flow diagram (Figure 1). A total of 5 clinical studies are included in this systematic review [16,17,18,19,20].

### 3.2. Study Characteristics

The first study by Husain M. et al. [16] (Table 1) assessed the effects of once-daily oral semaglutide 14 mg versus standard of care on patients with T2DM with established cardiovascular diseases (CVDs) or chronic kidney disease (CKD) or those who are 60 years old or more and only had cardiovascular risk factors. The treatment period was up to 83 weeks, with a dose escalation of 3 mg daily at the start of the study, followed by 7 mg daily at week 5, and 14 mg daily starting from week 9 until the end of the treatment period. The authors found statistically significant reductions in SBP (treatment difference of −2.6 (CI 95%: −3.7 to −1.5)) and DBP (treatment difference of 0.7 (95% CI: 0.0 to 1.3)). The authors also noted statistically significant reductions in TC, LDL, and TG in the treatment group, but the exact value was not reported. In contrast to these lipid profile results, HDL did not show a statistically significant change.

The second study from Pantanetti P. et al. [17] (Table 1), an observational prospective study, assessed the effects of oral semaglutide daily on patients ≥ 18 years old with T2DM. The intervention started with an initial dose of 3 mg oral semaglutide daily during the first month of the study, followed by 7 mg daily during the second month, and 14 mg from the third month until the end of the study period of six months. When comparing the post-semaglutide results at 6 months with the baseline results, the authors found statistically significant reductions in SBP (−12.74 mmHg; *p* < 0.05), DBP (−6.39 mmHg; *p* < 0.05), TC (−22.19 mg/dL; *p* < 0.05), LDL (−18.00 mg/dL; *p* < 0.05), and TG (−40.13 mg/dL; *p* < 0.05). The only outcome that did not show a statistically significant change was HDL (0.77 mg/dL; *p* = 0.31).

The third study by Aroda V.R. et al. [18] (Table 1) assessed the effects of oral semaglutide on the same patient population with T2DM receiving standard therapy. The authors compared the outcomes after oral semaglutide treatment for 68 weeks of the study period (starting with 3 mg daily for 4 weeks, escalating to 7 mg daily for another 4 weeks, and escalating to 14 mg daily the following weeks until the end of the study) with the pre-treatment values. The results of this study found a general trend in reductions in SBP (−4.2 mmHg; *p*-value not reported) and DBP (−2.4 mmHg; *p*-value not reported). The authors also noted a mild reduction in TC (ratio to baseline of 0.99; *p*-value not reported) and in TG (ratio to baseline of 0.82; *p*-value not reported). On the contrary, the trend in HDL and LDL change was a mild increase (ratio to baseline of 1.06 and 1.02, respectively; *p*-values were not reported for both HDL and LDL).

The fourth study by Volpe S. et al. [19] (Table 1) compared the effects of oral semaglutide after and before treatment on the same patient population that was 18 years old or older with T2DM. The length of the study was 26 weeks, with follow-ups after 3 months and 6 months following intervention. The authors assessed the effect of 7 mg of daily oral semaglutide at 26 weeks (with an initiation dose of 3 mg of daily oral semaglutide for 4 weeks, followed by 7 mg daily until the end of the study) as compared to patients’ baseline values (before the introduction of oral semaglutide) in SBP (128.7 ± 2.3 to 121.9 ± 2.6; *p* < 0.05), TC (154.4 ± 4.8 to 139.2 ± 3.6; *p* < 0.001), HDL (55.6 ± 2.5 to 51.6 ± 2.2; *p* < 0.05), and LDL (75.7 ± 4.2 to 65.7 ± 2.7; *p* < 0.05). Nevertheless, there was no significant change in DBP (74.9 ± 1.7 to 75.4 ± 1.7; *p* = NS) and TG (122.6 ± 12 to 111.6 ± 9.0; *p* = NS).

The last study by Lunati M.E. et al. [20] (Table 1) assessed the effects of oral semaglutide on patients who were 18 years old or older with T2DM who were taking dapagliflozin as a part of their T2DM treatment regimen. The authors evaluated the outcomes of patients with added oral semaglutide (mean dose of 12.76 mg/day) at the end of the 6-month study period as compared to those without oral semaglutide. They found statistically significant treatment differences across all five parameters: SBP (−5.4 ± 0.20; *p* < 0.0001), DBP (−4.30 ± 0.22; *p* < 0.0001), TC (−8.8 ± 0.5; *p* < 0.0001), HDL (+ 0.90 ± 0.12; *p* < 0.0001), LDL (−7.60 ± 0.49; *p* < 0.0001), and TG (+ 18.20 ± 0.82; *p* < 0.0001).

### 3.3. Quality of Studies

Quality assessment of studies in this review yielded good quality scores of 6 and above for cohort studies (Table 2) and of 5 and above for randomized controlled studies (Table 3).

## 4. Discussion

Our systematic review compiled data on cardiovascular risk factors for 5132 patients receiving oral semaglutide or placebo across five different, high-quality clinical studies available to this date.

### 4.1. Oral Semaglutide and Blood Pressure

The five studies included in this review seemed to report quite consistent results of oral semaglutide effects on SBP. Most studies reported a mild-to-moderate SBP reduction (−2.60 to −12.74 mmHg), with statistical significance (*p* < 0.05) in all but one [18]. Furthermore, such a reduction in SBP might also be clinically significant as 5 and 10 mmHg reductions in SBP are associated with a decreased risk of cardiovascular disease events by 10% and 20%, respectively [21].

In contrast to oral semaglutide’s effect on SBP, its effect on DBP varied between studies. Two studies (Pantanetti P. et al. and Lunati M.E. et al.) [17,20] demonstrated statistically significant reductions (*p* < 0.05) in DBP (−4.30 to −6.39 mmHg), and one study (Aroda V.R. et al.) [18] showed a trend in DBP reduction (−4.20 mmHg), while the other two studies (Husain M. et al. and Volpe S. et al.) [16,19] did not demonstrate statistically significant changes in DBP. Despite these differences, all five studies seem to agree that oral semaglutide does not raise DBP. Instead, the trend seems to point out that DBP is mildly affected (neutral-to-mild reduction).

Given its consistent SBP reduction and mild DBP effect, oral semaglutide may confer cardiovascular benefits.

The findings for SBP and DBP are consistent with Liakos C. et al. [22] who reviewed blood pressure-lowering effects on several types of GLP−1 RAs, including exenatide, liraglutide, dulaglutide, and semaglutide. The authors concluded that treatment with GLP-1 RAs resulted in a more consistent and sustained reduction in SBP, while their effect on DBP was less consistent. Their findings are also in line with the effect of oral semaglutide on DBP and SBP from the five studies mentioned in this review. Thus, this further increases our confidence in oral semaglutide’s blood pressure-lowering effect.

The mechanism by which GLP-1 RAs, including oral semaglutide, impact blood pressure is a topic of ongoing investigation. However, several researchers have tried to propose the mechanism by which GLP-1 RAs lower blood pressure. A review conducted by Ribeiro-Silva J. et al. [23] explained that GLP-1 RAs decrease blood pressure via two main possible mechanisms: modulation of the autonomic nervous system and vascular effects. Firstly, GLP-1 RAs decrease sympathetic activity both in the central and peripheral nervous systems. It is well known that increased sympathetic activity causes an increase in blood pressure. Thus, by counteracting sympathoexcitation, GLP-1 RAs lower blood pressure. Secondly, GLP-1 RAs have favorable effects on vascular physiology by regulating endothelial phenotype and increasing endothelial nitric oxide synthase (eNOS) as well as NO availability. All of these potentially contribute to improvement in endothelial function and vascular tone, resulting in a reduction in blood pressure.

The potential effect of GLP-1 RAs on NO physiology is also supported by Rowlands J. et al. [24]. Furthermore, the authors noted that GLP-1 RAs also prevent oxidative injury, especially endoplasmic reticulum stress and apoptosis induced by hyperglycemia, and inhibit the formation of macrophage foam cells in atherosclerotic plaque pathogenesis through various mechanisms [24,25]. These processes are what may potentially contribute to the improvement of blood pressure parameters (SBP and DBP) that can be observed across the study participants.

In addition to potential cardioprotection conferred by GLP-1 RAs’ effect on blood pressure parameters, the extravascular effect of GLP-1 RAs on cardiomyocytes is another proposed mechanism by which GLP-1 RAs improve cardiovascular health [26]. Cardiomyocytes express GLP-1R, and this may have cardioprotective effects by modulating pyroptosis.

### 4.2. Oral Semaglutide and Lipid Profile

The effects of oral semaglutide on lipid profiles vary for each specific parameter. The effect on total cholesterol is quite consistently reported by all five studies. All, except Aroda V.R. et al. [18], reported a statistically significant reduction in total cholesterol (*p* < 0.05) (ranging from −8.80 to −22.19 mg/dL) after taking oral semaglutide. Although Aroda V.R. et al. [18] did not report the statistical *p*-value for total cholesterol, their results showed a declining trend in total cholesterol (ratio to baseline after treatment: 0.99).

The change in LDL cholesterol shows a statistically significant decrease (*p* < 0.05) in all studies (ranging from −7.6 to −18.0 mg/dL), except in one study reported by Aroda V.R. et al. [18]. It is interesting to note that Aroda V.R. et al. [18] reported a slight increase in LDL cholesterol (ratio to baseline of 1.02), although it is unclear whether the increase in trend is statistically significant. Quite similar to oral semaglutide’s effect on LDL cholesterol, TG also shows a general decreasing trend in the studies (ranging from −11.00 to −40.13 mg/dL), except Lunati M.E. et al. [20] who reported an increase in TG (treatment difference +18.20 ± 0.82; *p* < 0.0001).

The exact mechanism of GLP-1’s impact on cholesterol metabolism remains under investigation. Nevertheless, GLP-1 inhibits the increase in postprandial triglyceride and free fatty acid in diabetic patients through delayed gastric emptying [25,27]. Additionally, studies have indicated that GLP-1 RAs are involved in several stages of cholesterol regulation, including the downregulation of lipogenic genes, the enhancement of lipolysis in human adipocytes, and the promotion of cholesterol efflux from foam cells via the ATP-binding cassette (ABCA1) transporter [27]. Another way to look at this lipid metabolism is through the lens of insulin and glucagon secretion. Activation of GLP-1R enhances insulin secretion and reduces glucagon secretion. Therefore, as suggested by Zheng Z. et al. [28], GLP-1 RAs can improve insulin signaling by reducing Akt phosphorylation and activating Protein Kinase C-ε, affecting the synthesis of triglycerides, phosphatidic acids, and diacylglycerols in the liver. These changes reduce the production of non-esterified fatty acids, glucose, and very-low-density lipoprotein (VLDL) synthesis.

In contrast to GLP-1 RA’s effect on total cholesterol, TG, and LDL, the effect on HDL cholesterol is the least consistently reported in all five studies. Three studies (Hussain M. et al., Pantanetti P. et al., and Aroda V.R. et al.) [16,17,18] reported no statistically significant change in HDL cholesterol, although the trend shows an increasing pattern. In addition, while Lunati M. et al. [20] reported a statistically significant increase in HDL (*p* < 0.0001), albeit at a very mild magnitude, Volpe S. et al. [19] reported a slight reduction in HDL instead. Thus, the effect in HDL seems to be less consistent. This finding is also demonstrated by several studies [29,30,31]. They used subcutaneous semaglutide, and it seems that their findings on HDL differ from each other. Even when a statistically significant change in HDL cholesterol is noted, whether a positive or a negative change, the mean treatment difference seems to be very mild, rendering the change in HDL value to be potentially insignificant clinically.

With regard to safety, the rates of adverse events were similar or markedly reduced in both groups. This finding is further corroborated by the findings in the study by Aroda V.R. et al. [18] which assessed the outcomes of patients receiving higher doses of semaglutide (up to 50 mg/day), wherein rates of adverse events were statistically or roughly similar to those in the lower dose group. This implies that oral semaglutide may have a wider-than-expected therapeutic index and that higher doses can be safely tolerated, although subgroup analyses with comorbidities and age groups must be studied.

After synthesizing the blood pressure and cholesterol metabolism findings, it is indicative that oral semaglutide may be cardioprotective. Its favorable effects on cardiovascular related parameters—blood pressure and non-HDL-cholesterol reducing effects—are postulated to decrease adverse cardiovascular events too given the time frame in which they were administered. This pharmacological profile is distinct from the rapid and more pronounced effect of SGLT2 inhibitors on heart failure, suggesting a possible synergy between the two drugs when used in combination.

### 4.3. Limitations of This Review

Despite these promising findings, we were not able to ascertain the different baseline treatment regimens provided to patients prior to and during oral semaglutide administration which present as confounding factors to the outcomes presented. Future studies could benefit from a uniform baseline treatment regimen or even comparisons of different baseline treatment regimens with or without oral semaglutide.

The same can be said about the comorbidities in patient profiles among studies as all studies included patients with various comorbidities such as hypertension, obesity, hyperlipidemia, etc., while there is no subgroup analysis for them. Future research should explore subgroup analysis to further ascertain the benefits of oral semaglutide on a more specific group of people.

Additionally, the included studies differ in sample size, especially between the observational prospective studies and randomized controlled trials. The former tends to have a smaller sample size than that of the latter. Despite the general discrepancy in the sample size between differing study designs, one observational prospective study by Lunati M.E. et al. [20] provided a comparable sample size for the treatment group to that of a randomized controlled study by Aroda V.R. et al. [18].

### 4.4. Recommendations for Future Studies

The implementation of oral semaglutide to existing T2DM treatment regimens could benefit from further data to support its use in clinical practice. Subgroup analyses comparing rates of adverse events between groups with different comorbidities, age groups, and BMIs would be helpful in determining risk versus benefit in different patient profiles. Comparisons with subcutaneous semaglutide could also be explored with regard to long-term compliance and patient convenience. The therapeutic synergy in the combination of oral semaglutide with SGLT2 inhibitors is also an appealing area to explore given both their cardioprotective effects. Prolonged duration of follow-up is also an area to potentially explore to evaluate efficacy and safety in longer-term administration. Although several studies exploring the underlying mechanism of semaglutide’s favorable effects on blood pressure, as well as its lipid profile, have been conducted, its direct cardiovascular protective effect is yet to be explored. Thus, such a study is also beneficial. With these additional findings, we believe it is only a matter of time before oral semaglutide will be pervasively found in T2DM patient treatment regimens worldwide.

Another parameter that semaglutide seemed to have improved compared to control/baseline groups is kidney outcomes. A meta-analysis by Sattar N. et al. mentioned that GLP-1 RAs reduced the risk of worsening kidney function by 18% (0.82 [0.69–0.98], *p* = 0.030) compared to 14% (0.86 [0.72–1.02], *p* = 0.089) in the control group [32]. The role of semaglutide on CKD progression continues to be studied, where it will be examined as a secondary outcome in an ongoing randomized controlled study (SOUL trial) with individuals with T2DM and established ASCVD and/or CKD as participants [21]. Positive findings with regard to renal function will further strengthen semaglutide’s place in diabetes treatment regimens worldwide.

## 5. Conclusions

The findings of this review demonstrated that regardless of randomized patient profiles across a variety of differently structured studies along with various comorbidities, age groups, and BMIs, once-daily oral semaglutide is a promising addition to the current T2DM treatment regimen in reducing cardiovascular risk factors, modifying disease parameters, and potentially reducing cardiovascular-related mortality in T2DM patients [18,20,21,32].

## Figures and Tables

**Table 1:** Study characteristics.

Study	Participants	Control	Therapy	Blood Pressure		Lipid Profile				Study Period
				SBP	DBP	Total C	HDL-C	LDL-C	TG	
**Husain M. et al. (2019)** [16] **Double-blind, randomized controlled trial**	Age ≥ 50 years old + established CVD/CKD, or age ≥ 60 years old + had CV risk factors only 3172 patients (1347 with oral semaglutide and 1435 with placebo)	Placebo + standard of care	Once-daily oral semaglutide 14 mg † + standard of care	Treatment difference (95% CI): −2.6 (−3.7; −1.5)	Treatment difference (95% CI): 0.7 (0.0; 1.3)	Statistically significant reduction at 50 weeks (exact value not reported)	No statistically significant change at 50 weeks	Statistically significant reduction at 50 weeks (exact value not reported)	Statistically significant reduction at 50 weeks (exact value not reported)	83 weeks
**Aroda VR. et al. (2023)** [18] **Double-blind randomized controlled trial**	Age ≥ 18 years with T2DM, HbA1c 8.0–10.5%, BMI ≥ 25.0 kg/m^2^, on stable daily doses of 1 to 3 of the following drugs: metformin, SU, SGLT2i, or DPP-4i At trial completion, the number of participants was 507 for 14 mg ‡	No control group Results are compared with baseline values as a part of treatment intensification	Once-daily oral semaglutide 14 mg †	Change from baseline: −4.2 mmHg (*p* = N.A.)	Change from baseline: −2.4 mmHg (*p* = N.A.)	Ratio to baseline: 0.99 (*p* = N.A.)	Ratio to baseline: 1.06 (*p* = N.A.)	Ratio to baseline: 1.02 (*p* = N.A.)	Ratio to baseline: 0.82 (*p* = N.A.)	68 weeks
**Volpe S. et al. (2023)** [19] **Prospective, open-label study**	Age ≥ 18 years old, T2DM 32 participants	No control group The results are compared with baseline values (on top of metformin)	Once-daily oral semaglutide 7 mg (starting 3 mg daily for the first 4 weeks, then escalating to 7 mg daily afterwards)	128.7 ± 2.3 to 121.9 ± 2.6 (*p* < 0.05).	74.9 ± 1.7 to 75.4 ± 1.7 (*p* = N.S.).	154.4 ± 4.8 to 139.2 ± 3.6 (*p* < 0.001)	55.6 ± 2.5 to 51.6 ± 2.2 (*p* < 0.05)	75.7 ± 4.2 to 65.7 ± 2.7 (*p* < 0.05)	122.6 ± 12 to 111.6 ± 9.0 (*p* = N.S.)	26 weeks
**Lunati ME. et al. (2024)** [20] **Observational, prospective study**	Age > 18 years old; T2DM; dapagliflozin or dapagliflozin + oral semaglutide. The final analysis includes 959 participants: 415 dapagliflozin and 544 dapagliflozin + semaglutide	Dapagliflozin + standard therapy (rapid-acting insulin, basal insulin, or SU/glinides)	Dapagliflozin + oral semaglutide (mean dose 12.76 mg/day) + standard therapy	* Treatment difference: −5.40 ± 0.20 (*p* < 0.0001)	* Treatment difference: −4.30 ± 0.22 (*p* < 0.0001)	* Treatment difference: −8.8 ± 0.5 (*p* < 0.0001)	* Treatment difference: +0.90 ± 0.12 (*p* < 0.0001)	* Treatment difference: −7.60 ± 0.49 *p* < 0.0001)	* Treatment difference: +18.20 ± 0.82 (*p* < 0.0001)	24 weeks (6 months)
**Pantanetti P. et al. (2024)** [17] **Observational prospective study**	Age ≥ 18 years old with T2DM The initial 100 participants and the final analysis includes 61 participants	No control group	Once-daily oral semaglutide 14 mg †	−12.74 mmHg; SD = 16.53; *p* < 0.05	−6.39 mmHg; SD = 12.04; *p* < 0.05	−22.19 mg/dL; SD = 46.26; *p* < 0.05	0.77 mg/dL; SD = 6.14; *p* = 0.31	−18.00 mg/dL; SD = 34.51; *p* < 0.05	−40.13 mg/dL; SD = 8.01; *p* < 0.05	24 weeks (6 months)

† Oral semaglutide initially started as 3 mg daily for the first 4 weeks, then escalated to 7 mg daily for the second 4 weeks, and finally to 14 mg daily afterwards. ‡ The study had 3 groups: once-daily oral semaglutide of 14 mg, 25 mg, and 50 mg. At trial completion, the number of participants was 507, 490, and 505, respectively. However, we only include the results from the 14 mg group in order to provide comparable treatment results with other studies included in this paper. * We ran our own *t*-test using the values given in the paper as the author did not calculate the treatment difference between the endpoints of dapagliflozin and dapagliflozin + semaglutide groups. N.A.; Not Available; N.S.; Not Significant.

**Table 2:** Quality assessment for cohort studies (Newcastle-Ottawa Scale).

No.	Study	Selection (Max *)				Comparability (Max **)	Outcome (Max *)			Score
		Representativeness of Exposed Cohort	Selection of Exposed Cohort	Ascertainment of Exposure	No Outcome of Interest at Start	Comparability of Cohorts Based on Design or Analysis	Assessment of Outcome	Was Followed Up Long Enough for Outcomes to Occur	Adequacy of Follow-Up of Cohorts	
1	Volpe S. et al., 2023 [19]	-	*	*	*	**	*	*	-	7
2	Pantanetti P. et al., 2024 [17]	-	*	*	*	**	*	*	*	8
3	Lunati M.E. et al., 2024 [20]	*	N.A.	*	-	*	*	*	*	6

N.A.; Not Applicable.

**Table 3:** Quality assessment for randomized controlled trial (Jadad scale).

No.	Study	Randomization		Blinding		Account of All Patients’	Score
		Randomization Mentioned	Appropriate Method of Randomization	Blinding Mentioned	Appropriate Method of Blinding	Fate (Withdrawn or Drop-Outs) of All Patients in Trial is Known	
1	Husain, M. et al., 2019 [16]	*	*	*	*	*	5
2	Aroda, V.R. et al., 2023 [18]	*	*	*	*	*	5

## Data Availability

The original contributions presented in this study are included in the article. Further inquiries can be directed to the corresponding author.

## Abbreviations

The following abbreviations are used in this manuscript:

ABCA1	ATP-binding cassette
ASCVD	Atherosclerotic cardiovascular disease
BMI	Body mass index
CI	Confidence interval
CKD	Chronic kidney disease
CVD	Cardiovascular disease
DBP	Diastolic blood pressure
eNOS	Endothelial nitric oxide synthase
GLP-1	Glucagon-like peptide-1
GLP-RA	Glucagon-like peptide-1 receptor agonist
HDL	High-density lipoprotein
LDL	Low-density lipoprotein
NOS	Newcastle-Ottawa Scale
PRISMA	Preferred Reporting Items for Systematic Reviews and Meta-Analyses
SBP	Systolic blood pressure
SGLT2	Sodium glucose cotransporter 2
T2DM	Type 2 diabetes mellitus
TC	Total cholesterol
TG	Triglyceride
VLDL	Very-low-density lipoprotein
